# Development and Implementation of a Mobile Tool for High-Risk Pregnant Women to Deliver Effective Caregiving for Neonatal Abstinence Syndrome: Protocol for a Mixed Methods Study

**DOI:** 10.2196/27382

**Published:** 2021-04-15

**Authors:** Ekaterina Burduli, Hendrée E Jones, Olivia Brooks, Celestina Barbosa-Leiker, Ron Kim Johnson, John Roll, Sterling Marshall McPherson

**Affiliations:** 1 College of Nursing Washington State University Spokane, WA United States; 2 Analytics and PsychoPharmacology Laboratory Washington State University Spokane, WA United States; 3 UNC Horizons, Department of Obstetrics and Gynecology School of Medicine University of North Carolina at Chapel Hill Chapel Hill, NC United States; 4 Managed Health Connections, LLC Spokane, WA United States; 5 Elson S. Floyd College of Medicine Washington State University Spokane, WA United States

**Keywords:** neonatal abstinence syndrome, opioid use disorder, mHealth, maternal child outcomes

## Abstract

**Background:**

The United States continues to experience an alarming rise in opioid use that includes women who become pregnant and related neonatal abstinence syndrome (NAS) in newborns. Most newborns experiencing NAS require nonpharmacological care, which entails, most importantly, maternal involvement with the newborn. To facilitate positive maternal-newborn interactions, mothers need to learn effective caregiving NAS strategies when they are pregnant; however, an enormous gap exists in the early education of mothers on the symptoms and progression of NAS, partly because no education, training, or other interventions exist to prepare future mothers for the challenges of caring for their newborns at risk for NAS.

**Objective:**

In this paper, we describe a mixed methods, multistage study to adapt an existing mobile NAS tool for high-risk pregnant women and assess its usability, acceptability, and feasibility in a small randomized controlled trial.

**Methods:**

Stage 1 will include 20 semistructured interviews with a panel of neonatology experts, NAS care providers, and mothers with experience caring for NAS-affected newborns to gather their recommendations on the management of NAS and explore their perspectives on the care of these newborns. The findings will guide the adaptation of existing mobile NAS tools for high-risk pregnant women. In stage 2, we will test the usability, acceptability, and feasibility of the adapted mobile tool via surveys with 10 pregnant women receiving opioid agonist therapy (OAT). Finally, in stage 3, we will randomize 30 high-risk pregnant women receiving OAT to either receive the adapted mobile NAS caregiving tool or usual care. We will compare these women on primary outcomes—maternal drug relapse and OAT continuation—and secondary outcomes—maternal-newborn bonding; length of newborn hospital stays; readmission rates; breastfeeding initiation and duration; and postpartum depression and anxiety at 4, 8, and 12 weeks postpartum.

**Results:**

This project was funded in July 2020 and approved by the institutional review board in April 2020. Data collection for stage 1 began in December 2020, and as of January 2021, we completed 18 semistructured interviews (10 with NAS providers and 8 with perinatal women receiving OAT). Common themes from all interviews will be analyzed in spring 2021 to inform the adaptation of the NAS caregiving tool. The results from stage 1 are expected to be published in summer 2021. Stage 2 data collection will commence in fall 2021.

**Conclusions:**

The findings of this study have the potential to improve NAS care and maternal-newborn outcomes and lead to commercialized product development. If effective, our new tool will be well suited to tailoring for other high-risk perinatal women with substance use disorders.

**Trial Registration:**

ClinicalTrials.gov NCT04783558; https://clinicaltrials.gov/ct2/show/NCT04783558

**International Registered Report Identifier (IRRID):**

DERR1-10.2196/27382

## Introduction

### Background

The United States is experiencing an alarming rise in opioid use during pregnancy, diagnosis of opioid use disorder (OUD) in pregnancy, and related neonatal abstinence syndrome (NAS) in newborns [1,2]. The percentage of pregnant women reporting opioid misuse increased from 2% to 28% in the United States from 2000 to 2014, and the proportion of pregnant women who entered substance use treatment and reported prescription opioids as their primary substance increased from 16.9% in 1996 to 41.6% in 2014 [3]. NAS is a spectrum of symptoms or signals of substance withdrawal in newborns whose mothers used illicit opioids or were being treated with opioid agonist therapy (OAT; ie, methadone or buprenorphine) during pregnancy [4,5]. NAS rates have dramatically increased from approximately 1.5 to 8.0 per 1000 hospital births from 2004 to 2014 [5-8], which translates to one infant experiencing opioid withdrawal every 15 minutes in the United States [1]. There is evidence that this rate continues to increase as the US Pediatric Health Information System reported an incidence of 20 per 1000 live births in 2016 [9]. NAS signs include tremors, increased irritability, fever, sweating, extreme weight loss, excessive sucking, and inability to sleep [4,10,11]. NAS is an enormously costly public health issue, costing the United States US $563 million in an aggregate associated hospital charge in 2014, largely because of costs associated with lengthy hospital stays, neonatal intensive care unit (NICU) admissions, and higher risk of hospital readmission, and other costly, downstream health costs associated with depression, anxiety, and other difficulties that mothers encounter [1,6,12,13].

Mothers are often unprepared for what they are about to experience before having a baby while using either illicit or licit opioids. Treatment of NAS in the postpartum period is critical in shaping both maternal and newborn outcomes. At least half [14] of the newborns experiencing NAS require nonpharmacological care, which is best delivered by the newborn’s mother or close caregiver [4,15-17]. Up to 95% of NICUs offer some form of nonpharmacological intervention, and it is advised that all infants with in utero exposure to opioids be administered nonpharmacological treatment [14]. Evidence-based, nonpharmacological care for NAS includes rooming together with the mother postdelivery and modification of the environment to support maternal-newborn attachment that promotes a soothing environment for the infant. Specific actions include low-stimulating environments (reduced noise and light), frequent and small feedings, swaddling, promotion of breastfeeding, and continual contact and soothing from the caregiver [15,17-20]. These are all doable now by mothers and other health care providers; however, the missing link is having ready access to an educational platform to promote these strategies, which is precisely what this study is designed to do.

Facilitating successful postpartum maternal-newborn involvement is critical in lowering NICU admissions and hospital readmissions, boosting breastfeeding, shortening hospital stays, decreasing the need for pharmacological intervention for the newborn, reducing the risk of maternal OAT discontinuation, and facilitating maternal-child bonding [13,15,21-24]. However, the challenging NAS signs can hinder maternal-newborn bonding, particularly for women who have substance use disorders (SUDs) who may have difficulty responding to newborns’ cues [15]. To maximize positive maternal-newborn interactions, mothers need to learn effective NAS caregiving strategies when they are pregnant to optimize their preparedness; however, little to no preparedness strategies exist for at-risk pregnant women.

The *American College of Obstetricians and Gynecologists’ Alliance for Innovation on Maternal Health Program* recently developed a maternal safety bundle on obstetric care of women with OUDs, which emphasizes the need to provide caregivers education regarding NAS and newborn care [19]. However, pregnant women in OAT currently receive virtually zero education or consultation for what to expect when they give birth to a baby who is at risk of experiencing NAS. One such education strategy would incorporate caregiving skills based on the promising and recently established Eat, Sleep, and Console (ESC) model that stresses newborn sleep, feeding, and a low-stimulating environment [20]. We previously developed and implemented a mobile instructional tool designed to streamline the assessment of NAS for providers in the NICU. This interactive tool includes evidence-based education modules covering topics such as NAS epidemiology, symptoms, nonpharmacological treatment, and transition to follow-up care [25].

### Objectives

**Figure 1:**
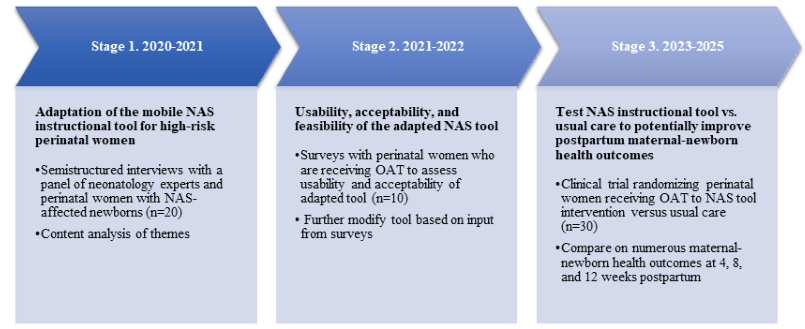
Stage process and time line of study. NAS: neonatal abstinence syndrome; OAT: opioid agonist therapy.

As the long-term caregivers of newborns experiencing NAS, mothers are essential to the successful, nonpharmacological treatment of NAS-affected babies. By adapting our instructional NAS tool, we can address this critical gap and potentially improve outcomes for NAS-affected newborns and their mothers. This study describes a mixed methods, multi-stage study (see Figure 1 for study stage and timeline) to adapt an existing mobile NAS tool for high-risk pregnant women and assess its usability, acceptability, and feasibility in a small randomized controlled trial.

## Methods

### Overview

This study will be carried out in 3 stages. In stage 1, we will conduct semistructured interviews with a panel of NAS care experts and mothers with NAS-affected newborns until saturation is reached (expected n=20) to document their perspectives and gather their recommendations on the care of newborns with NAS. Although findings from stage 1 will ultimately guide the adaptation of the existing mobile NAS tool for high-risk pregnant women, the skills training in the adapted tool will be largely based on elements of ESC. In stage 2, we will test the usability, acceptability, and feasibility of the adapted mobile tool via surveys with 10 pregnant women receiving OAT at 2 facilities in Washington: one that provides methadone as its pharmacotherapy and another that primarily treats patients receiving buprenorphine as their pharmacotherapy. In stage 3, we will randomize 30 high-risk pregnant women seen at these facilities to either receive the adapted mobile NAS instructional tool or usual care. We will compare these participants on numerous maternal and newborn health outcomes at 4, 8, and 12 weeks postpartum, including *primary outcomes*—maternal OAT continuation and maternal drug relapse—and *secondary outcomes*—length of newborn hospital stay, readmission rates, maternal-newborn bonding, breastfeeding initiation and duration, birth satisfaction, and postpartum depression and anxiety.

### Adapting the Mobile NAS Tool for High-Risk Pregnant Women

All videos in the existing modules for NICU providers have a script, with different content covered in each video. We will modify these modules (eg, titles, scripts, and content reading levels) to increase their relevance for high-risk pregnant women and for nonexperts. We expect that the adaption process will require (1) the removal of existing educational modules that are not appropriate for at-risk pregnant women (eg, *Epidemiology and Pathophysiology of NAS*), (2) reduction of video lengths, and (3) development of modules based on qualitative findings that are appropriate for high-risk pregnant women (eg, ESC-based caregiving skills for mothers, postpartum maternal resources, preparing for hospital length of stay, and preparing for possibly experiencing health care stigma). For example, one existing module is titled *Epidemiology and Pathophysiology of NAS*, which provides considerable detail and medical terminology. We expect that this module will need to be substantially reduced and only include a brief overview of this topic in a way that is appropriate for at-risk pregnant women. We also expect that the adapted tool will include brand-new educational modules and accompanying videos that visually demonstrate ESC-based caregiving skills that are known to soothe a NAS-affected newborn and prevent further complications. We expect that the total number of modules for perinatal women will not exceed 6.

After this initial process of modification, the modules will be uploaded to a private account on YouTube, and all participating individuals (ie, NAS providers and mothers with NAS-affected babies) will be given free web access (via a YouTube link) to the existing modules and will have the opportunity to review the existing content before providing feedback on further necessary changes and modifications. They will be asked to provide their opinion on the relevance of a new audience (pregnant women in treatment for OUD). Once semistructured interviews are completed and common themes are analyzed using qualitative description methodology, we will schedule a mini conference with the participants of this study stage (ie, NAS care experts and mothers with NAS-affected babies) to discuss the interpretation of the qualitative findings and double check for consistency that the planned adaptation of the mobile NAS tool matches what the participants tried to convey.

Although findings will ultimately guide the adaptation of the existing mobile NAS tool for high-risk pregnant women, the skills training in the adapted tool will be largely based on elements of ESC. Therefore, we anticipate that the NAS mobile tool intervention will incorporate nonintrusive caregiving skills and strategies that encompass providing a low-stimulating environment (eg, dimmed light and low noise), swaddling, continuous comfort and contact with the caregiver, skin-to-skin contact, frequent feeding, and novel components identified in the interviews.

### Stage 1: Adaptation of the Mobile NAS Instructional Tool for Pregnant Women at Risk for Having Newborns With NAS

#### Setting and Data Collection

We will recruit 10 pregnant and postpartum women from 2 locations: a regional health district’s opioid treatment program and local recovery center to participate in the semistructured interviews. Recruitment materials with contact information for study staff and a link to a brief information statement that describes the screening process and eligibility requirements will be distributed to the 2 programs via email and presented at staff meetings to recruit the 10 pregnant and/or postpartum participants. At least 10 NAS care experts (broadly defined as any person providing NAS services such as, but not limited to, nurses, social workers, obstetricians and gynecologists, and neonatologists) will also be identified and contacted from existing partnerships with local and national hospitals. After a verbal or PowerPoint description of the adapted module topics and content is provided and participants have a chance to ask questions, we will ask participants to provide feedback on the module topics and content (eg, “What do you think of the modules?” “Do you think you could use it with relative ease? What are any challenges to using it?” “Are any topics missing from the modules? “Do you think this would help you care for a baby with Neonatal Abstinence Syndrome after birth? If yes, how? If no, what could improve it?” “What do you think we should add to this study?” “Any additional thoughts and suggestions about the modules?”). Each participant who completes the interview will receive a US $25 Amazon e-gift card for their time and an additional US $25 Amazon gift card for participation in a miniconference to discuss qualitative findings. All interviews will be conducted via video or phone conferences.

#### Inclusion Criteria

Eligibility criteria were selected to allow for an accurate assessment of challenges to NAS care and necessary evidence-based skills to care for NAS-affected newborns, while maximizing generalizability of results. Inclusion criteria for NAS care experts are (1) NAS providers with significant expertise in NAS care, (2) age >18 years, and (3) ability to speak and understand English. The inclusion criteria for pregnant and postpartum participants are (1) a pregnant woman currently in OAT for OUD or a postpartum woman who has experience caring for NAS-affected newborns, (2) age >18 years, and (3) ability to speak and understand English. Participants not meeting the eligibility criteria will be excluded.

#### Data Analysis

All interviews will be digitally audio recorded and then transcribed verbatim by a professional transcriptionist. The deidentified interview transcriptions will be uploaded into NVivo version 11, a software program produced by QSR International for qualitative research. The qualitative description methodology described by Schreier [26] will be used to analyze the data. A qualitative description methodology is used when the goal is to summarize descriptions of events or experiences in a way that depicts the perspectives of the participants [27,28]. Common themes will be identified in the data (transcripts) to provide definitions and details of the most prominent ideas provided by participants’ responses. The qualitative description methodology will involve combing concept-driven and data-driven analysis approaches to the text; reliability will be addressed by the process of having each researcher (ie, principal investigator, research coordinator, and research team) initially review and analyze the data before each meeting and then comparing consistency of agreement between the researchers, and validity will be addressed by considering the applicability of the themes when compared with participants’ responses. An audit trail will be kept throughout the analysis process to document decisions and next steps.

### Stage 2: Usability, Acceptability, and Feasibility of the Adapted NAS Tool

#### Data Collection and Outcomes

We will recruit 10 pregnant women receiving OAT from 2 locations: a regional health district’s opioid treatment program and a local recovery center to participate in the survey, as described earlier in stage 1. Consented participants will be asked to review the adapted content (via a private YouTube link) before completing the surveys. Once they review the adapted modules, they will be directed to complete a Research Electronic Data Capture survey that will assess several measures. Acceptability will be examined by the 8-item Client Satisfaction Questionnaire (CSQ-8) to rate the overall satisfaction with the adapted NAS tool [29]. Usability will be assessed using the 10-item Systems Usability Scale (SUS), in which they are asked to answer questions about a mobile app (eg, “I think that I would need the support of a technical person to be able to use this app”) [30,31]. To assess feasibility, a measure of utility will be assessed via several questions (eg, “To what extent do you expect to be able to incorporate the NAS caregiving tool in your daily activities during pregnancy and postpartum?”), tracking how many times participants referred to specific modules within the mobile tool, and open-ended questions that ask participants to comment on the feasibility of this tool along with overall impressions and comments on the tool (survey details are given in Tables 1 and 2). Participants will receive a US $25 e-gift card for their time.

**Table 1:** Measures and timing of data collection for stage 3 randomized controlled trial.

Outcomes		Baseline (during the third trimester)	4, 8, and 12 weeks postpartum
**Demographic characteristics**			
	Age	✓^a^	N/A^b^
	Education level	✓	N/A
	Marital status	✓	N/A
	Time in OAT^c^ program	✓	N/A
	Number and age of living children	✓	N/A
	Employment	✓	N/A
**Baseline characteristics**			
	Addiction Severity Index-Lite	✓	N/A
	Maternal Antenatal Attachment Scale	✓	N/A
**Outcome measures**			
	Addiction Severity Index-Lite	✓	✓
	Maternal Postpartum Attachment Scale	N/A	✓
	Birth Satisfaction Scale-Revised	N/A	✓
	Patient Health Questionnaire-9	N/A	✓
	Parenting Stress Index	N/A	✓
	Client Satisfaction Questionnaire-8	N/A	✓
	System Usability Scale	N/A	✓
	Length of hospital stay for newborn	N/A	✓
	Newborn hospital readmission	N/A	✓
	Breastfeeding initiation and duration	N/A	✓
	Maternal OAT continuation and relapse	N/A	✓
	Frequency of NAS^d^ tool use (weekly)	✓	✓

^a^Data collected.

^b^N/A: not applicable.

^c^OAT: opioid agonist therapy.

^d^NAS: neonatal abstinence syndrome.

**Table 2:** Outcomes and description of measures.

Study stage and outcome			Study design		Description of measures
**Stage 1: NAS^a^ tool adaptation**					
	Tool adaptation	Semistructured interviews		Semistructured interviews with a panel of NAS care experts and mothers with NAS-affected newborns until we reach saturation (expected n=10) to document their perspectives and gather their recommendations on the care of newborns with NAS.	
**Stage 2:** **Usability, acceptability, and feasibility of the adapted NAS tool**					
	Usability	Survey		Participants will complete a 10-item SUS^b^, in which they are asked to answer questions about a mobile app (eg, “I think that I would need the support of a technical person to be able to use this app”) using a 5-point Likert scale, ranging from strongly disagree to strongly agree. The SUS is currently the industry standard for evaluation of a wide variety of products and services such as software, mobile devices, websites, and apps [30,31]. The SUS has been shown to successfully differentiate between usable and unusable systems, and it can be used in small samples with reliable results [30,31].	
	Acceptability	Survey		Acceptability will be examined by the CSQ-8^c^ to rate the overall satisfaction with the adapted NAS tool [29]. CSQ-8 is a validated questionnaire measuring satisfaction with health services (eg, “How would you rate the quality of care your received?”) that has been translated in more than 30 languages [29,32,33]. Possible total scores on CSQ-8 range from 8 to 32, with higher scores (>23) indicating greater satisfaction with health services.	
	Feasibility	Survey		To assess feasibility, a measure of utility will be assessed (eg, “To what extent do you expect to be able to incorporate the NAS caregiving tool in your daily activities during pregnancy and postpartum?”), tracking how many times participants referred to specific modules within the mobile tool and open-ended questions that ask participants to comment on feasibility of this tool along with overall impressions and comments.	
**Stage 3:** **Clinical trial comparing NAS tool with usual care**					
	Demographic characteristics	Randomized clinical trial		Demographics and baseline characteristics, such as age, education, marital status, time in the OAT^d^ program, number and age of living children, employment, and ASI-Lite^e^, will be collected at the start of the study to describe the sample and to serve as control variables when comparing groups on the outcomes.	
	Maternal drug use and relapse	Randomized clinical trial		Maternal drug use and relapse will be assessed via ASI-Lite, a standardized semistructured clinical interview that offers clinical information and assesses severity profiles in the following domains: medical, employment, alcohol, drug, psychological, legal, and family and social [34]. It has been shown to have adequate to good internal consistency, good test-retest reliability, independence across the domain composite scores, and agreement with the longer version of the ASI-Lite [34].	
	OAT continuation	Randomized clinical trial		OAT continuation will be assessed via a single question (“Are you currently receiving OAT (Yes or No)? Please explain”).	
	Length of newborn hospital stay and readmission	Randomized clinical trial		These will be determined by single questions: length of newborn hospital stay (“how many days did your newborn stay in the hospital”) and newborn hospital readmission (“has your newborn been readmitted to the hospital for any reason after discharge? If yes, how many times? Please list reasons for each readmission”).	
	Perinatal maternal-fetal attachment	Randomized clinical trial		Prenatal maternal-fetal attachment will be measured with the Maternal Antenatal Attachment Scale [35,36], a reliable and validated 19-item measure of maternal-fetal attachment that includes several response formats and assesses 2 dimensions: 1) quality of attachment (11 items) and 2) intensity of preoccupation (8 items). The total scores range from 19 to 95, with higher scores indicating a higher level of attachment to the fetus [35-39]. Maternal-newborn bonding will be measured via MPAS^f^ [40,41]. It consists of 19 items assessing 3 dimensions: *pleasure in interaction with the infant* (5 items), *absence of hostility toward the infant* (5 items), and *quality of mother-infant attachment* (9 items). Response categories range from 2-, 3-, 4-, to 5-point scales, for different items. The total score ranges from 19 to 95, with higher scores indicating higher maternal postpartum attachment to the baby. The MPAS has been found to have an acceptable level of reliability (Cronbach α ranging from .75 to .79; test-retest reliability *r*=0.86; *P*<.001) [37,40,41].	
	Maternal birth experience	Randomized clinical trial		The Birth Satisfaction Scale-Revised, a 10-item, Likert-type birth satisfaction questionnaire that measures experiences of childbearing, stress, quality of care, and women’s attributes, was psychometrically validated in the United States by our research team [42-44]. Its response categories range from 1=strongly disagree to 5=strongly agree, with higher scores indicating higher birth satisfaction.	
	Maternal Depression	Randomized clinical trial		Assess via the PHQ-9^g^, a psychometrically validated 9-item measure used to assess depression in a variety of populations [45-47]. The PHQ-9 asks participants to report on the degree they were bothered by 9 symptoms over the past 2 weeks (ie, “little interest or pleasure in doing things” and “feeling down, depressed, or hopeless”), with response categories ranging from 0=not at all to 3=nearly every day and higher scores indicating greater levels of depression symptomology.	
	Maternal stress	Randomized clinical trial		PSI^h^ measures parental stress associated with the perception of having a difficult child or a dysfunctional parent-child relationship and consists of 36 items that are rated on a 5-point Likert scale (from 1=strongly agree to 5=strongly disagree), with higher scores indicative of less total stress [48]. PSI has been shown to possess good psychometric properties and has been validated in numerous samples, including high-risk families [49]. It includes parental distress, parent-child dysfunctional interaction, and difficult child subscales, all of which will be considered individually. The child and parent domains can and will be combined to form a total stress scale score.	
	Breastfeeding	Randomized clinical trial		Assessed via several questions “Are you currently breastfeeding? If yes, ‘How often do you breastfeed your baby?’, if no, ‘How long did you breastfeed your baby.’”	
	Frequency of NAS tool use	Randomized clinical trial		Throughout their third trimester and postpartum, we will also send participants a brief weekly web-based survey link asking about the previous week’s frequency of use of the adapted mobile NAS tool and which specific modules participants viewed most, if any, to track weekly frequency of use and preference of modules.	

^a^NAS: neonatal abstinence syndrome.

^b^SUS: Systems Usability Scale.

^c^CSQ-8: 8-item Client Satisfaction Questionnaire.

^d^OAT: opioid agonist therapy.

^e^ASI-Lite: Addiction Severity Index-Lite.

^f^MPAS: Maternal Postpartum Attachment Scale.

^g^PHQ-9: 9-item Patient Health Questionnaire

^h^PSI: Parenting Stress Index short form.

#### Inclusion Criteria

Participants will be pregnant women (1) who are in the third trimester currently in OAT treatment for OUD, (2) aged >18 years, and (3) who are able to speak and understand English. Participants who did not meet the eligibility criteria will be excluded.

#### Data Analysis

Descriptive analyses will be conducted using percentages, means, and SDs to describe the feasibility and acceptability of the adapted mobile NAS tool for pregnant women receiving OAT. All descriptive analyses will be conducted using STATA version 14.2. This developmental stage will not require limited inferential tests because the sample sizes are small, and the qualitative work will be the primary driver of the modifications to be made and eventually tested in a randomized trial in stage 3.

### Stage 3: Clinical Trial to Test the NAS Instructional Tool Versus Usual Care to Potentially Improve Postpartum Maternal-Newborn Health Outcomes

#### Data Collection and Inclusion Criteria

Similar to previous stages, we will recruit 30 pregnant women in their third trimester from the same 2 treatment centers to participate in stage 3 of the study. After a brief phone screening, eligible participants will then be consented and randomized 1:1 into either the intervention condition (ie, adapted NAS tool intervention, as given in the *Study Intervention* section) or the control condition (ie, treatment as usual [TAU], as given in the *Study Intervention* section). Participants will be asked to provide demographic and baseline information at this point. Women in the intervention condition will go through the mobile-based NAS instructional tool at least once during pregnancy, with their choice of going through the modules gradually while waiting at the OAT clinic to receive their dose or by scheduling a time to review the modules with research staff outside of the clinic (either at university offices or via a video conference). Participants will be met with a research assistant who will provide them with an iPad and access to the modules (via a private YouTube link), and the research assistant will be available for any questions throughout the review. Participants will be able to create a YouTube account on the iPad and will be able to access, skip, pause, and continue the modules at any time during the duration of the study through the 12-week follow-up period (ie, access after giving birth as well). They will also be able to post questions or comments under private YouTube videos for research staff to see and address. Each participant will also be scheduled for the 3 follow-up appointments (at 4, 8, and 12 weeks postpartum). Follow-up appointments will consist of filling out surveys (Table 1). Participants will be able to complete the survey on their phones or iPads. For completing all study appointments, participants will receive a total of US $125 in Amazon e-gift cards (US $25 after providing demographic and baseline information, US $25 after reviewing the modules with a research assistant, and an additional US $25 Amazon gift card for each of the 3 follow-ups). The inclusion criteria are as follows: (1) a pregnant woman currently undergoing OAT treatment for OUD, (2) age >18 years, and (3) ability to speak and understand English. Exclusion criteria are as follows: recurring (eg, daily or almost daily) thoughts of harming themselves or others in the past 2 weeks.

#### Study Intervention

TAU pregnant women in this condition will receive care as usual, which involves continued enrollment in OAT and continued obstetric care. We will also provide them with a printed handout containing information on NAS and local resources. This level of information meets or exceeds what most mothers in this situation usually receive. Participants in the TAU condition will not receive iPads with accompanying modules; however, the handout constitutes more information than they normally receive.

#### Adapted NAS Tool Intervention

Pregnant women in this condition will receive the adapted mobile-based NAS instructional tool and TAU. Women in this condition will go through the NAS instructional tool at least once during pregnancy, with their choice of going through the modules gradually while waiting at the OAT clinic to receive their dose or by scheduling a time to review the modules. Participants will have free web access to the tool throughout their third trimester and through 12 weeks postpartum so they can access the modules at any time and as many times as desired, including after giving birth. Women will be randomized 1:1 to the intervention or TAU conditions.

#### Data Collection and Outcomes

Although the primary outcomes of focus will be maternal drug relapse (assessed via Addiction Severity Index-Lite) and OAT continuation (“Are you currently receiving OAT (Yes or No)? Please explain*.*”), because of the exploratory nature of this trial, several other outcomes of interest will also be assessed. At the 3 follow-up appointments, participants will complete several measures assessing numerous maternal-newborn outcomes: the 9-item Patient Health Questionnaire, a psychometrically validated 9-item measure used to assess depression in a variety of populations [45-47]; the *Birth Satisfaction Scale-Revised*, a 10-item, Likert-type, birth satisfaction questionnaire that measures experiences of childbearing, stress, quality of care, and women’s attributes and that was psychometrically validated in the United States by our research team [42-44]; the *Parenting Stress Index short form*, which measures parental stress associated with the perception of having a difficult child or a dysfunctional parent-child relationship and consists of 36 items [48]; and the *Maternal Postpartum Attachment Scale (MPAS)* [40,41], which measures maternal-newborn bonding. The *MPAS* consists of 19 items assessing 3 dimensions: *pleasure in interaction with the infant* (5 items), *absence of hostility toward the infant* (5 items), and *quality of mother-infant attachment* (9 items), with higher scores indicating higher maternal postpartum attachment to the baby [37,40,41]. We will also collect other outcome measures, including the length of newborn hospital stay (“How many days did your newborn stay in the hospital”), newborn hospital readmission (“Has your newborn been readmitted to the hospital for any reason after discharge? If yes, how many times? Please list reasons for each readmission.”), and breastfeeding (“Are you currently breastfeeding? If yes, ‘How often do you breastfeed your baby?’ if no, ‘How long did you breastfeed your baby?’”). Acceptability and satisfaction of the NAS tool will also be examined at follow-up by the CSQ-8 to rate overall satisfaction with the adapted NAS tool [29]. Finally, participants will also complete the *10-item SUS* [30,31] (Table 2). Throughout their third trimester and postpartum, we will also send participants a brief weekly web survey link asking about the previous week’s frequency of use of the adapted mobile NAS tool and which specific modules participants viewed the most, if any, to track weekly frequency of use and preference of modules.

#### Data Analysis

Means (SDs) will be calculated for continuous variables, and percentages will be calculated for categorical variables for the 2 groups (intervention group and TAU group) at each assessment. Demographic and baseline characteristics will be tested across intervention versus TAU via independent-samples *t* tests and one-way analysis of variance (for continuous variables) and chi-square tests (for categorical variables). Generalized estimating equations will be used to analyze the primary longitudinal outcomes of maternal drug relapse and OAT continuation and secondary outcomes and to control for any baseline demographic differences across groups, where intervention versus TAU remains as the primary independent variable. Analyses will control for baseline and demographic outcomes and time. All inferential results will be presented as odds ratios with 95% CIs for binary outcomes and unstandardized regression coefficients with 95% CIs for continuous outcomes. We will use an α error rate of .05 as the threshold for statistical significance. All analyses will be conducted using STATA version 14.2.

## Results

This project was funded in July 2020 (see Multimedia Appendix 1 for grant review summary statement) and approved by the institutional review board in April 2020. Data collection for stage 1 began in December 2020, and as of January 2021, we completed 18 semistructured interviews (10 with NAS providers and 8 with perinatal women receiving OAT). Common themes from all interviews will be analyzed in spring 2021 to inform the adaptation of the NAS caregiving tool. Results from stage 1 are expected to be published in summer 2021. Stage 2 data collection will commence in fall 2021, followed by the randomized controlled trial in stage 3 in late 2022.

## Discussion

### Conclusions

This study is highly innovative in several ways. First, despite the need to educate pregnant women at risk for delivering NAS-affected newborns and equip them with the skills necessary to successfully care for a newborn with NAS when they are pregnant, no published evidence-based interventions exist to prepare future mothers of potential NAS-affected babies. This study will be the first to adapt an existing mobile NAS tool and develop an intervention for use with high-risk pregnant women and may be the first NAS-related intervention strategy designed to be administered to pregnant women in an OAT setting. Second, if effective, the mobile nature of our new tool will be readily scalable and well suited to tailoring for other populations of high-risk women (ie, with alcohol and or SUDs) of reproductive age. Moreover, such a tool will also be easy to update and modify, as more evidence emerges for how best to treat NAS. Although the tool is still too new to evaluate its cost efficiency, this study will prepare this tool for future analysis in the context of a larger study.

### Strengths and Limitations

The scope of the project is unavoidably limited. First, the relatively small number of sites and pregnant women participating in stage 3 hinders generalizability and the ability to determine effectiveness. However, this novel and critically relevant trial capitalizes on existing investments to allow us to gather data on efficacy and inform a fully powered randomized controlled trial. Second, recruitment below expectation and attrition are always possible. If recruitment becomes difficult, we will hold problem-solving meetings with program staff and research team on recruitment and study advertisement strategies. However, given our proposed study sample of n=30, we foresee no issue with obtaining our target enrollment across the 2 recruitment sites. Third, although attrition can be high in high-risk populations, our participants will already be embedded within the OAT system and, therefore, already engaged with services.

All activities outlined in the proposed application to develop and evaluate the mobile NAS caregiving intervention are accompanied with enhancing and evaluating the contextual fit (eg, acceptability and appropriateness) of the NAS caregiving intervention to support the application and scalability of this tool. This new tool will be designed to remain flexible to novel scientific breakthroughs in this domain such that new and modified modules can be easily created and integrated into this unique educational platform.

## Appendix

Multimedia Appendix 1 Grant review summary statement from the NIH.

## Abbreviations

CSQ-8: 8-item Client Satisfaction Questionnaire
ESC: Eat, Sleep, and Console
MPAS: Maternal Postpartum Attachment Scale
NAS: neonatal abstinence syndrome
NICU: neonatal intensive care unit
OAT: opioid agonist therapy
OUD: opioid use disorder
SUD: substance use disorder
SUS: Systems Usability Scale
TAU: treatment as usual
