# Supplementary material for: Development and Implementation of a Mobile Tool for High-Risk Pregnant Women to Deliver Effective Caregiving for Neonatal Abstinence Syndrome: Protocol for a Mixed Methods Study
Source: JMIR Res Protoc. 2021 Apr 15;10(4):e27382. doi: 10.2196/27382 (PMC8085745; doi:10.2196/27382)
Supplement: Multimedia Appendix 1 [file resprot_v10i4e27382_app1.pdf]

SUMMARY STATEMENT
(Privileged Communication)

PROGRAM CONTACT:
KEISHER Highsmith
301-402-1984
keisher.highsmith@nih.gov

Release Date: 03/09/2020
Revised Date:

Application Number: 1 K01 DA051780-01
Formerly: 1K01NR019295-01

Principal Investigator

BURDULI, EKATERINA

Applicant Organization: WASHINGTON STATE UNIVERSITY

Review Group: CMPC
Clinical Management of Patients in Community-based Settings Study Section

Meeting Date: 02/10/2020
Council: MAY 2020
Requested Start: 07/01/2020
RFA/PA: PA19-127
PCC: CM/KHS

Project Title: Effective Caregiving for Neonatal Abstinence Syndrome: Development of an Instructional Mobile Technology Platform for High-Risk Pregnant Women
SRG Action: Impact Score:23 Percentile:6 +
Next Steps: Visit https://grants.nih.gov/grants/next_steps.htm
Human Subjects: 30-Human subjects involved - Certified, no SRG concerns
Animal Subjects: 10-No live vertebrate animals involved for competing appl.
Gender: 2A-Only women, scientifically acceptable
Minority: 1A-Minorities and non-minorities, scientifically acceptable
Age: 3U-No children included, scientifically unacceptable

Project Year	Direct Costs Requested	Estimated Total Cost
1	146,463	158,180
2	149,239	161,178
3	153,743	166,042
4	158,298	170,962
5	162,659	175,672
TOTAL	770,402	832,034

ADMINISTRATIVE BUDGET NOTE: The budget shown is the requested budget and has not been adjusted to reflect any recommendations made by reviewers. If an award is planned, the costs will be calculated by Institute grants management staff based on the recommendations outlined below in the COMMITTEE BUDGET RECOMMENDATIONS section.

1K01DA051780-01 Burduli, Ekaterina

COMMITTEE BUDGET RECOMMENDATIONS
INCLUSION ACROSS THE LIFESPAN PLAN UNACCEPTABLE

RESUME AND SUMMARY OF DISCUSSION: This applicant is seeking a mentored career development award to support the goal of becoming an independent investigator with an established program of research focused on the development, implementation, and testing of interventions for substance abuse among perinatal women and reducing poor health outcomes for women and their newborns. The panel described this candidate as excellent, with a productive history of funding and publications and with previous research in birth outcomes and substance use disorders. The candidate's collaborators represent the key disciplines necessary to support the research, and there is an established relationship with the mentor, including prior publications. The career development goals are clearly articulated and additional training in substance abuse intervention in pregnant women and in the administration of clinical trials will support the candidate's career development. Strengths in the research plan include the scientific justification for the proposed project is sound given that opioid use disorder and neonatal abstinence syndrome are significant issues. The delivery of the educational intervention through a mobile tool is innovative and scalable. Utilizing two study sites for recruitment increases the feasibility and generalizability. Reviewers also noted minor and addressable weaknesses, notably a justification for the exclusion of those under 18 would strengthen the study design. Overall, this application will have a high impact on developing a research program to reduce poor birth outcomes among substance using perinatal women and their newborns.

DESCRIPTION (provided by applicant): Due to an alarming rise in opioid use among the general population that is mirrored in pregnant women, Neonatal Abstinence Syndrome (NAS) rates have increased in the US from 2004 to 2014. Most newborns experiencing NAS require non-pharmacologic care, which entails, most importantly, maternal involvement with her newborn. Facilitating postpartum maternal-newborn involvement is critical in preventing further adverse maternal-newborn outcomes. To achieve positive maternal-newborn involvement, mothers need to learn effective caregiving NAS strategies while they are pregnant. Surprisingly, current obstetrical practice standards for high risk pregnant women do not address this pressing need, in part because no interventions exist to prepare future mothers for the challenges of caring for their newborns at risk for NAS. To address this critical gap, I propose to adapt an existing mobile NAS tool for clinician training and decision support, for high-risk pregnant women and assess its usability, acceptability, and feasibility in a small randomized controlled analog trial. First, I will conduct semi- structured interviews with a panel of neonatology experts, NAS care providers, and mothers with NAS-affected babies to gather their recommendations on management of NAS and explore their perspectives on the care of these newborns. Findings will guide the adaptation of the existing mobile NAS tool for high-risk pregnant women. I will then test the usability, acceptability, and feasibility of the adapted mobile tool via surveys with 10 pregnant women receiving opioid agonist therapy (OAT) at Spokane Regional Health District's Opioid Treatment Program and Evergreen Recovery Center. Finally, we will randomize 30 high-risk pregnant women seen at these facilities to either receive the adapted mobile NAS caregiving tool or usual care. We will compare these mothers on maternal drug relapse and OAT continuation, maternal-newborn bonding, length of newborn hospital stays, readmissions rates, breastfeeding initiation and duration, and postpartum depression and anxiety at 4, 8, and 12 weeks postpartum. Findings will serve as pilot data for a subsequent large R01 randomized controlled analog trial testing the efficacy of the adapted NAS caregiving tool in reducing poor outcomes for NAS-affected newborns and their mothers. My proposed research plan integrates activities, formal training, and mentorship from experts (Drs. Sterling McPherson, Hendree Jones, John Roll, Celestina Barbosa-Leiker, and Kim Johnson) in development, testing and implementation of substance use disorder treatment for perinatal women, mobile health interventions, and implementation of clinical trials in perinatal women with substance use disorders. This Mentored Research Scientist Development Award (K01) will build upon my previous training and

allow me to pursue my long-term career goal of becoming an independent investigator with an established program of research focused on the development, implementation, and testing of interventions for substance using perinatal women and reduction of poor health outcomes for substance using perinatal women and their newborns.

PUBLIC HEALTH RELEVANCE: Most newborns experiencing Neonatal Abstinence Syndrome (NAS) require non-pharmacologic care, which entails, most importantly, maternal involvement with her newborn. To facilitate positive maternal-newborn interactions, mothers need to learn effective caregiving NAS strategies while they are pregnant, yet, an enormous gap exists in the early education of mothers on the symptoms and progression of NAS, in part because no interventions exist to prepare future mothers for the challenges of caring for their newborns at risk for NAS. In this project, I propose to adapt an existing mobile NAS tool for high-risk pregnant women and assess its usability, acceptability, and feasibility in a small randomized controlled analog trial, and engage in career development and training activities that will enhance my expertise in the development, implementation, and testing of interventions for substance using perinatal women.

CRITIQUE 1

Candidate: 1
Career Development Plan/Career Goals /Plan to Provide Mentoring: 4
Research Plan: 5
Mentor(s), Co-Mentor(s), Consultant(s), Collaborator(s): 2
Environment Commitment to the Candidate: 2

Overall Impact: This career development award application is considered to be high impact due to the excellent qualifications of the candidate, a PhD psychologist with a research background in birth satisfaction and birth outcomes as well as a postdoc in substance use disorders and health disparities, the complementary expertise of an esteemed mentorship team with an established track record of collaboration and mentorship, and the clearly delineated training plan and candidate goals. The candidate has been extremely productive and articulates her commitment to the research area and her future plans clearly. Several minor concerns regarding the research plan in terms of the approach to adaptation of the existing tool and the ability to obtain data to inform a future grant proposal from the proposed study as well as potential concerns about the fact that the second mentor is located across the country slightly weaken enthusiasm for the application, but the qualifications of the candidate and the team remain extremely compelling.

1. Candidate

Strengths

- The candidate has been extremely productive in terms of funding and publications during her postdoctoral fellowship (17 manuscripts, 2 PI grants and multiple Co-I).
- The candidate provides a strong justification for how she identified her area of interest and how her future work will build on her prior experiences.
- Despite her impressive track record, additional training in SUD interventions in pregnant women and administration of clinical trials will be helpful in her career development.

Weaknesses

- None noted by reviewer.

2. Career Development Plan/Career Goals & Objectives

Strengths

- The goals are well articulated and clearly tied to plans for future research and grant applications.

Weaknesses

- Additional description of what the Clinical Trials Certificate training entails would be helpful.
- Additional exposure to the administration of randomized trials through the mentorship team would better prepare the candidate for a future grant application.
- While there is excellent statistical mentorship, additional coursework may be useful (depending on the content of the clinical trials certificate).
- The second portion of qualitative training seems to occur after the qualitative portion of the study is completed.

3. Research Plan

Strengths

- The scientific justification for the proposal is sound: OUD and NAS are significant problems and involving mothers in the care of the newborn is optimal but requires education. Prior qualitative work by the candidate demonstrates that mothers do not feel they are receiving appropriate education.
- Delivering the educational intervention via a mobile- based educational intervention is innovative and potentially scalable.
- The existence of a tool for providers that can be modified for mothers increases the feasibility of the study.
- Utilizing 2 study sites for recruitment improves feasibility and increases generalizability.

Weaknesses

- Reference is made to consulting a “qualitative expert” at WSU; if this is (or is not) Dr. Barbosa-Leiker, they should be identified.
- Significant modification of the existing tool is needed, which may increase expense and requires significant time, which is appropriately reflected in the time allotted for initial development. However, if this is to be modified based on the information from the qualitative work at the end of Aim 1 and in Aim 2, the timeline may need to be adjusted.
- Given the expected duration of NAS/implementation of ESC (frequently under a week, in most cases up to a month) the rationale for additional postpartum follow up could be further justified.
- Small sample size limits the ability to perform statistical analyses in Aim 2 and Aim 3, particularly regarding clinical outcomes. Though no power calculation is performed for this reason, this should be addressed in the proposal to delineate how feasibility and acceptability data can be obtained in preparation for a future grant application. A larger sample size seems feasible based on the timeline and would likely provide better grounding for a future grant application in this area.
- Similarly, implementing strategies for dealing with missing data may be challenging based upon the small sample size.

4. Mentor(s), Co-Mentor(s), Consultant(s), Collaborator(s)

Strengths

- The primary mentor, Dr. McPherson, is experienced in biostatistics, clinical trial methodology, technology development and addiction treatment and education development, the areas that the

candidate needs training in. They have an established mentorship relationship and have published together.

- Dr. Jones is a leader in the field of interventions in pregnant and postpartum women with substance use disorder and an experienced mentor.
- Dr. Barbosa-Leiker has experience in qualitative research and can serve as a professional mentor. She has a longstanding mentorship relationship with the candidate.
- The WSU team have a track record of collaboration and a commitment to methodologic expertise.
- The mentorship team is complementary in their skills and experience.

Weaknesses

- Dr. Jones is located at UNC. Though there is a plan to address the lack of physical proximity and her unique expertise is directly relevant to the proposed project and the candidate's career goals, additional plans could be put in place if this mentorship at a distance proves challenging.

5. Environment and Institutional Commitment to the Candidate

Strengths

- The resources offered by WSU, the Program of Excellence in Addiction Research, access to the NIDA clinical trials network and the partnerships with the Spokane Regional Health District and the Evergreen Recovery Center will provide an outstanding environment for the candidate.
- There is a prior track record of collaboration between the partner organizations and the mentorship team.
- Candidate has been promoted to a tenure track position.

Weaknesses

- None noted by reviewer.

Study Timeline

Strengths

- Appropriate time for recruitment for the randomized trial

Weaknesses

- Additional time may be needed for adaptation of the educational tool prior to the RCT. Though the proposed timeline is reasonable, the sample size may be too small to be informative.

Protections for Human Subjects

Acceptable Risks and Adequate Protections

- Appropriate protections for a vulnerable population.

Data and Safety Monitoring Plan (Applicable for Clinical Trials Only):

Acceptable

- Data Safety Monitoring Plan in place

Inclusion Plans

- Sex/Gender: Distribution justified scientifically
- Race/Ethnicity: Distribution justified scientifically

- Inclusion/Exclusion Based on Age: Distribution justified scientifically

Vertebrate Animals

Not Applicable (No Vertebrate Animals)

Biohazards

Not Applicable (No Biohazards)

Training in the Responsible Conduct of Research

Acceptable

Comments on Format (Required):

- CITI training, WSU ethics seminars, and mentorship meetings

Comments on Subject Matter (Required):

- CITI training will be the most targeted training with regards to topic matter.

Comments on Faculty Participation (Required; not applicable for mid- and senior-career awards):

- Faculty are involved through mentorship meetings

Comments on Duration (Required):

- Sustained throughout the award

Comments on Frequency (Required):

- Monthly seminars unless integrated into mentorship meetings.

Select Agents

Not Applicable (No Select Agents)

Resource Sharing Plans

Not Applicable (No Relevant Resources)

Budget and Period of Support

Recommended budget modifications or possible overlap identified:

- It is not clear why 30 iPads are needed if 30 people are to be randomized and half will receive usual care.

CRITIQUE 2

Candidate: 1
Career Development Plan/Career Goals /Plan to Provide Mentoring: 2
Research Plan: 3
Mentor(s), Co-Mentor(s), Consultant(s), Collaborator(s): 1
Environment Commitment to the Candidate: 1

Overall Impact*:* The proposed K01 career development and research plan has a very high likelihood to enhance the candidate's potential for a productive, independent scientific research career in the research areas of perinatal substance use disorder and NAS prevention. This project is especially timely given the perinatal opioid epidemic. The project addresses a critical gap in perinatal SUD research, prevention of NAS. Innovation and rigor are strengths in this application due to use of emerging mHealth technologies that are scalable/replicable.. The mentoring team consists of national experts in NAS, perinatal substance use disorder and biostatistics. Given the candidates life experience, prior work and professional goals, Dr. Burduli is well matched with an expert mentoring team highly capable of providing leadership and expertise needed to successfully implement the research plan. Dr. Burduli is an outstanding candidate with an ambitious research plan. Score driving factors include candidate's prior work and publication record, innovation using mHealth with RCT design, expert mentoring team and clearly defined research project and data analysis plan. I am highly confident Dr. Burduli will be successful in meeting her career and research goals; thus significantly advancing the science of perinatal substance use disorder prevention and treatment.

1. Candidate

Strengths

- Dr. Burduli has relevant, previous training with an expert mentoring team focused on development, implementation, interventional research in both substance using perinatal women and substance exposed newborns.
- Postdoctoral fellowship experience with application and award of pilot funding work with a large scale, state-wide datasets; and service as a Co-Principal Investigator NIAAA's Native Center for Alcohol Research and Education Program and Pilot Project
- Over the past 3-5 years, Dr. Burduli has had a strong record of publications and dissemination relevant to the proposed work.

Weaknesses

- None noted by reviewer.

2. Career Development Plan/Career Goals & Objectives

Strengths

- Dr. Burduli's proposes three career goals centered on gaining research experience in the the areas of perinatal addiction/NAS, mobile health technology research methods (RCT). Each career goal is well defined, with reasonable objectives.
- Each career goal clearly matches with the expertise of the proposed mentoring/consulting team.
- Given current and former collaborations among the current members of the proposed mentoring team, there is high likelihood the candidate will have a high level of engagement in both 1:1 and K01 team meetings.

Weaknesses

- The wide breadth of training goals; though all relevant to the candidate's research area of interest, are ambitious given the time. Each of the three career goals could potentially stand-alone.

3. Research Plan

Strengths

- The study aims address a highly significant and growing national issue, perinatal SUD and NAS
- Innovation is high with use of mHealth, operationalized in the prenatal period for prevention of NAS.

- Use of a mixed methods design incorporating both qualitative methods (providers and pregnant/postpartum women) and quantitative methods provides a deeper context to help guide adaptation of the health intervention.
- Data analysis plan is appropriate.

Weaknesses

- For mHealth intervention justification of exclusion of age (<18) would strengthen design.
- For mHealth intervention there is need to account for recruited pregnant women who may experience a miscarriage and/or lethal fetal event.
- Eligibility of pregnant women is limited and does not specify gestational-weeks of pregnancy, parity and/or inclusion of multifetal gestation; thus justification with regard to the proposed broad inclusion criteria would strengthen inclusion criteria.

4. Mentor(s), Co-Mentor(s), Consultant(s), Collaborator(s) 1

Strengths.

- Dr. McPherson is a national expert in her field, and has demonstrated a strong publication and dissemination record relevant to the candidate's professional goals, including leading RCT's in opioid use disorder treatment, experimental models of addiction treatment and behavioral pharmacology behavioral and pharmacotherapeutic addiction treatment. (training goals 1-3).
- Dr. Jones is a distinguished, national expert in perinatal addiction and NAS that has relevant experience to guide candidate on work with both pregnant women as well as their infants. Further, she is the executive director of the national benchmark for residential treatment for pregnant and parenting women (Horizons, training goal 1).
- Over the past 5 years, Dr. Barbosa-Leiker (consultant) has had significant publications in both qualitative and quantitative methods and is well poised to provide oversight/mentorship for training goal 1.
- For the past several years, Drs. McPherson, Barbosa-Leiker and Roll have current evidence of significant collaboration via professional and published work, focused on a vast array of addiction research, including but not limited to contingency management interventions targeting alcohol and tobacco use disorders, data analysis and interpretation of qualitative/quantitative results (training goals 1-3).
- Dr. Johnson's is a leading expert in healthcare innovation (mhealth) via creation of decision tools, received the INTM Healthcare Innovation Challenge CMMI grant, and has recent experience as PI for a NINA-funded NAS Reference and Decision Support Tool, highly relevant expertise to assist Dr. Buldoil (training goal 2).

Weaknesses

- It was difficult to discern the publication and dissemination record of Dr. Johnson.

5. Environment and Institutional Commitment to the Candidate

Strengths

- The environment at Spokane is outstanding due to the institutional commitment to research in substance use treatment (PEAR: a program to advance behavioral and pharmacotherapeutic interventions for addiction), bioinformatics center and proven record of support to community partners offering substance use treatment.
- The presence of highly successful programs/centers, Spokane Regional Health District's Opioid Treatment Program and Evergreen Recovery Center (enthusiastic letters of support included).

Weaknesses

- None noted by reviewer.

Study Timeline

Strengths

- Efficiently detailed to meet project milestones

Weaknesses

- None noted by reviewer.

Protections for Human Subjects

Acceptable Risks and Adequate Protections

Data and Safety Monitoring Plan (Applicable for Clinical Trials Only):

Acceptable

Inclusion Plans

- Sex/Gender: Distribution justified scientifically
- Race/Ethnicity: Distribution justified scientifically
- Inclusion/Exclusion Based on Age: Distribution not justified scientifically
- Justification of <18 not provided

Vertebrate Animals

Not Applicable (No Vertebrate Animals)

Biohazards

Not Applicable (No Biohazards)

Training in the Responsible Conduct of Research

Acceptable

Comments on Format (Required):

Comments on Subject Matter (Required):

Comments on Faculty Participation (Required; not applicable for mid- and senior-career awards):

Comments on Duration (Required):

Comments on Frequency (Required):

Select Agents

Not Applicable (No Select Agents)

Resource Sharing Plans

Acceptable

Budget and Period of Support

Recommend as Requested

CRITIQUE 3

Candidate: 1
Career Development Plan/Career Goals /Plan to Provide Mentoring: 1
Research Plan: 5
Mentor(s), Co-Mentor(s), Consultant(s), Collaborator(s): 4
Environment Commitment to the Candidate: 1

Overall Impact: This K application is from a junior investigator at Washington State University School of Nursing. She is a strong junior investigator with a productive track record and a well described program of research. The Career development plan is strong and is aligned with the development of the candidate. The institutional commitment to the candidate is excellent. The Approach has a number of moderate weaknesses. A large concern is related to the small proposed study for the approach and its unlikely to provide adequate data for a future R01-level study. The mentorship plan has some weaknesses with no career mentor and there is no plan for career mentorship which would strengthen this application.

1. Candidate

Strengths

- This is a strong researcher who has been very productive in her career to date publishing extensively and seeking strong connections and mentorship.

Weaknesses

- None noted by reviewer.

2. Career Development Plan/Career Goals & Objectives

Strengths

- Strong career development plan
- Appropriate coursework is proposed aligned with research aims.

Weaknesses

- None noted by reviewer.

3. Research Plan

Strengths

- Iterative design process
- Mixed methods approach

Weaknesses

- Framework for developing the mHealth tool would increase the rigor and reproducibility of the findings
- SUS is not specific to mHealth technology

- Usability assessment is not included; rigorous methods are needed to ensure the usability of the tool
- Given that this is a 5 year K, I would expect the trial to be more rigorous and include a longer timeframe and/ or a larger sample.
- A sample of only 30 participants who will be randomized to separate arms has the potential to result in minimal pilot data for any future work.

4. Mentor(s), Co-Mentor(s), Consultant(s), Collaborator(s)

Strengths

- The mentors have relevant experience and expertise and have a strong plan to support the candidate

Weaknesses

- A mentorship team of 2 mentors is smaller than what one would expect for a Kaward
- Further the mentors provide domain expertise but a career mentor is also important
- Minor (one of the two mentors is located in NC).

5. Environment and Institutional Commitment to the Candidate

Strengths

- There is very strong institutional commitment to this candidate
- Environment provides the resources and clinical environment to support the proposed work.

Weaknesses

- None noted by reviewer.

Study Timeline

Strengths

- The proposed activities are definitely achievable in 5 years

Weaknesses

- Timeline is very generous for the scope of work and training proposed.

Protections for Human Subjects

Acceptable Risks and Adequate Protections

Data and Safety Monitoring Plan (Applicable for Clinical Trials Only):

Acceptable

Inclusion Plans

- Sex/Gender: Distribution justified scientifically
- Race/Ethnicity: Distribution justified scientifically
- Inclusion/Exclusion Based on Age: Distribution justified scientifically

Vertebrate Animals

Not Applicable (No Vertebrate Animals)

Biohazards

Not Applicable (No Biohazards)

Training in the Responsible Conduct of Research

Acceptable

Comments on Format (Required):

- Plans to re-take the CITI course

Comments on Subject Matter (Required):

- The subject matter in the CITI training is acceptable

Comments on Faculty Participation (Required; not applicable for mid- and senior-career awards):

- The faculty will be participating in the training

Comments on Duration (Required):

- The duration of the training is acceptable but can certainly be more robust

Comments on Frequency (Required):

- This appears to be a one-time training; more frequent training me be valuable.

Resource Sharing Plans

Not Applicable (No Relevant Resources)

Budget and Period of Support

Recommend as Requested

THE FOLLOWING SECTIONS WERE PREPARED BY THE SCIENTIFIC REVIEW OFFICER TO SUMMARIZE THE OUTCOME OF DISCUSSIONS OF THE REVIEW COMMITTEE, OR REVIEWERS' WRITTEN CRITIQUES, ON THE FOLLOWING ISSUES:

PROTECTION OF HUMAN SUBJECTS: ACCEPTABLE

INCLUSION OF WOMEN PLAN: ACCEPTABLE

INCLUSION OF MINORITIES PLAN: ACCEPTABLE

INCLUSION ACROSS THE LIFESPAN PLAN: UNACCEPTABLE

- Justification of <18 not provided

COMMITTEE BUDGET RECOMMENDATIONS:

- It is not clear why 30 iPads are needed if 30 people are to be randomized and half will receive usual care.

Footnotes for 1 K01 DA051780-01; PI Name: Burduli, Ekaterina

+ Derived from the range of percentile values calculated for the study section that reviewed this application.

NIH has modified its policy regarding the receipt of resubmissions (amended applications).See Guide Notice NOT-OD-18-197 at https://grants.nih.gov/grants/guide/notice-files/NOT-OD-18-197.html. The impact/priority score is calculated after discussion of an application by averaging the overall scores (1-9) given by all voting reviewers on the committee and multiplying by 10. The criterion scores are submitted prior to the meeting by the individual reviewers assigned to an application, and are not discussed specifically at the review meeting or calculated into the overall impact score. Some applications also receive a percentile ranking. For details on the review process, see http://grants.nih.gov/grants/peer_review_process.htm#scoring.

MEETING ROSTER

Clinical Management of Patients in Community-based Settings Study Section
Healthcare Delivery and Methodologies Integrated Review Group
CENTER FOR SCIENTIFIC REVIEW
CMPC
02/10/2020 - 02/11/2020

Notice of NIH Policy to All Applicants: Meeting rosters are provided for information purposes only. Applicant investigators and institutional officials must not communicate directly with study section members about an application before or after the review. Failure to observe this policy will create a serious breach of integrity in the peer review process, and may lead to actions outlined in NOT-OD-14-073 at https://grants.nih.gov/grants/guide/notice-files/NOT-OD-14-073.html and NOT-OD-15-106 at https://grants.nih.gov/grants/guide/notice-files/NOT-OD-15-106.html, including removal of the application from immediate review.

CHAIRPERSON(S)

HODGSON, NANCY A, PHD
ASSOCIATE PROFESSOR
DEPARTMENT OF BIOBEHAVIORAL HEALTH SCIENCES
SCHOOL OF NURSING
UNIVERSITY OF PENNSYLVANIA
PHILADELPHIA, PA 19104

MEMBERS

ABEL, WILLIE M, PHD *
ASSOCIATE PROFESSOR
SCHOOL OF NURSING
UNIVERSITY OF NORTH CAROLINA - CHARLOTTE
CHARLOTTE, NC 28223

ANDERSON, JOEL G, PHD *
ASSOCIATE PROFESSOR
COLLEGE OF NURSING
UNIVERSITY OF TENNESSEE
KNOXVILLE, TN 37996

APARASU, RAJENDER R, PHD
PROFESSOR AND CHAIR
DEPARTMENT OF PHARMACEUTICAL
HEALTH OUTCOMES AND POLICY
COLLEGE OF PHARMACY, TEXAS MEDICAL CENTER
UNIVERSITY OF HOUSTON
HOUSTON, TX 77204

ASHFORD, KRISTIN H, PHD *
PROFESSOR
COLLEGE OF NURSING
UNIVERSITY OF KENTUCKY
LEXINGTON, KY 40536

BADGER, TERRY A, RN, PHD
PROFESSOR
DEPARTMENT OF NURSING
COLLEGE OF NURSING
UNIVERSITY OF ARIZONA
TUCSON, AZ 85721

BELANGER, EMMANUELLE, PHD, MS, BA *
ASSISTANT PROFESSOR
SCHOOL OF PUBLIC HEALTH
BROWN UNIVERSITY
PROVIDENCE, RI 02912

DUNN, SUSAN L, BSN, MSN, PHD *
ASSOCIATE PROFESSOR
DEPARTMENT OF BIOBEHAVIORAL HEALTH SCIENCE
COLLEGE OF NURSING
UNIVERSITY OF ILLINOIS AT CHICAGO
CHICAGO, IL 60612

DWIBEDI, NILANJANA, PHD *
ASSISTANT PROFESSOR
SCHOOL OF PHARMACY
WEST VIRGINIA UNIVERSITY
MORGANTOWM, WV 26506

EAKIN, MICHELLE NUTTALL, PHD *
ASSOCIATE PROFESSOR
DIVISION OF PULMONARY AND CRITICAL
CARE MEDICINE
SCHOOL OF MEDICINE
JOHNS HOPKINS UNIVERSITY
BALTIMORE, MD 21287

EVANGELISTA, LORRAINE S, PHD, RN *
PROFESSOR
SCHOOL OF NURSING
UNIVERSITY OF CALIFORNIA IRVINE
LOS ANGELES, CA 90095

FAN, VINCENT S, MD, MPH
ASSOCIATE PROFESSOR
DEPARTMENT OF MEDICINE
VA PUGET SOUND HEALTH CARE SYSTEM
HEALTH SERVICES RESEARCH AND DEVELOPMENT
UNIVERSITY OF WASHINGTON
SEATTLE, WA 98108

FOWLER, NICOLE R, PHD, MHSA *
ASSISTANT PROFESSOR OF MEDICINE
CENTER FOR AGING RESEARCH
SCHOOL OF MEDICINE
INDIANA UNIVERSITY
INDIANAPOLIS, IN 46022

GIBSON, ROBERT WILLIAM, PHD
PROFESSOR AND DIRECTOR OF RESEARCH
DEPARTMENT OF EMERGENCY MEDICINE
MEDICAL COLLEGE OF GEORGIA
AUGUSTA UNIVERSITY
AUGUSTA, GA 30912

GRIFFIN, JOAN M, PHD *
ASSOCIATE PROFESSOR
HEALTH SCIENCES RESEARCH
MAYO CLINIC COLLEGE OF MEDICINE
ROCHESTER, MN 55905

HAQUE, REINA, PHD
SENIOR RESEARCH SCIENTIST III
DEPARTMENT OF RESEARCH AND EVALUATION
KAISER PERMANENTE SOUTHERN CALIFORNIA
PASADENA, CA 91101

HUDSON, TERESA JO, PHMD, PHD *
PROFESSOR OF PSYCHIATRY
CENTER OF HEALTH SERVICES RESEARCH
PSYCHIATRIC RESEARCH INSTITUTE
UNIVERSITY OF ARKANSAS FOR MEDICAL SCIENCES
LITTLE ROCK, AR 72205

HWANG, SUNAH S, MD, BA, MPH, PHD *
ASSISTANT PROFESSOR
PEDIATRICS-NEONATOLOGY
UCHEALTH UNIVERSITY OF COLORADO HOSPITAL
AURORA, CO 80045

JACELON, CYNTHIA S, PHD, RN, FAAN *
PROFESSOR
DEPARTMENT OF NURSING, SCHOOL OF NURSING
UNIVERSITY OF MASSACHUSETTS
AMHEREST, MA 01003

KAIMAL, ANJALI, MAS, AB, MD *
ASSOCIATE PROFESSOR
DEPARTMENTS OF OBSTETRICS, GYNECOLOGY
AND REPRODUCTIVE BIOLOGY AND POPULATION MEDICINE
HARVARD MEDICAL SCHOOL
BOSTON, MA 02114

KALPAKJIAN, CLAIRE ZABELLE, AB, MS, PHD *
ASSOCIATE PROFESSOR
DEPARTMENT OF PHYSICAL MEDICINE
AND REHABILITATION
UNIVERSITY OF MICHIGAN
ANN ARBOR, MI 48109

KEEFER, LAURIE A, PHD *
PROFESSOR
DEPARTMENT OF MEDICINE-GASTROENTERLOGY
AND PSYCHIATRY
ICAHN SCHOOL OF MEDICINE AT MOUNT SINAI
NEW YORK, NY 10029

KEIM -MALPASS, JESSICA KEIM, BS, MS, MSN, PHD *
ASSISTANT PROFESSOR
ASSISTANT PROFESSOR OF PEDIATRICS
SCHOOL OF MEDICINE
UNIVERSITY OF VIRGINIA SCHOOL OF NURSING
CHARLOTTESVILLE, VA 22908

KEMPER, ALEX R, MD, MPH
PROFESSOR
DEPARTMENT OF PEDIATRICS
NATIONWIDE CHILDREN'S HOSPITAL
THE OHIO STATE UNIVERSITY
COLUMBUS, OH 43205

LINDQUIST, LEE A, MD, MPH *
ASSOCIATE PROFESSOR
FEINBERG SCHOOL OF MEDICINE
NORTHWESTERN UNIVERSITY
CHICAGO, IL 60611

MAGWOOD, GAYENELL SMITH, FAAN, PHD
PROFESSOR
DEPARTMENT OF NURSING
COLLEGE OF NURSING
MEDICAL UNIVERSITY OF SOUTH CAROLINA
CHARLESTON, SC 29425

MCELFISH, PEARL, MBA, PHD *
VICE-CHANCELLOR NORTHWEST ARKANSAS CAMPUS
COLLEGE OF NURSING
UNIVERSITY OF ARKANSAS FOR MEDICAL SCIENCES
FAYETTEVILLE, AR 72703

MCMAHON, SIOBHAN KATHLEEN, PHD, GNPBC, MPH *
ASSOCIATE PROFESSOR
SCHOOL OF NURSING
UNIVERSITY OF MINNESOTA
MINNEAPOLIS, MN 55455

NELSON, LONNIE A, BA, MA, PHD *
ASSISTANT PROFESSOR
DEPARTMENT OF NURSING
WASHINGTON STATE UNIVERSITY
SEATTLE, WA 98105

NESS, KIRSTEN KIMBERLIE, PHD
FULL MEMBER
DEPARTMENT OF EPIDEMIOLOGY AND CANCER CONTROL
SAINT JUDE CHILDREN'S RESEARCH HOSPITAL
MEMPHIS, TN 38105

PIATT, GRETCHEN A, PHD
ASSOCIATE PROFESSOR
DEPARTMENT OF LEARNING HEALTH SCIENCES
UNIVERSITY OF MICHIGAN
ANN ARBOR, MI 48109

SATAGOPAN, JAYA M, PHD *
PROFESSOR
DEPARTMENT OF BIOSTATISTICS AND EPIDEMIOLOGY
SCHOOL OF PUBLIC HEALTH
RUTGERS UNIVERSITY
PISCATAWAY, NJ 08854

SCHNALL, REBECCA, MBA, MPH, RN, PHD *
MARY DICKEY LINDSAY ASSOCIATE PROFESSOR
HEALTH PROMOTION AND DISEASE PREVENTION
SCHOOL OF NURSING
COLUMBIA UNIVERSITY
NEW YORK, NY 10032

STEVENS, ALAN B, PHD *
PROFESSOR AND VICE CHAIR FOR RESEARCH
CENTENNIAL CHAIR IN GERONTOLOGY
DEPARTMENT OF INTERNAL MEDICINE
TEXAS A&M UNIVERSITY
TEMPLE, TX 76508

SUN, VIRGINIA CHIH-YI, BS, MSN, PHD, RN *
ASSOCIATE PROFESSOR
DIVISION OF NURSING RESEARCH AND EDUCATION
DEPARTMENT OF POPULATION SCIENCES
CANCER CONTROL AND POPULATION SCIENCES PROGRAM
CITY OF HOPE
DUARTE, CA 91010

SZALACHA, LAURA A, EDD
PROFESSOR
RESEARCH METHODOLOGY AND BIOSTATISTICS
MORSANI COLLEGE OF MEDICINE
UNIVERSITY OF SOUTH FLORIDA
TAMPA, FL 33612

TUROK, DAVID, MD, MPH
ASSOCIATE PROFESSOR
DEPARTMENT OF OBSTETRICS AND GYNECOLOGY
UNIVERSITY OF UTAH
SALT LAKE CITY, UT 84132

VRANCEANU, ANA-MARIA, PHD *
ASSOCIATE PROFESSOR
DEPARTMENT OF PSYCHIATRY
MASSACHUSETTS GENERAL HOSPITAL
BOSTON, MA 02114

WANG, DONGWEN, PHD
PROFESSOR
DEPARTMENT OF BIOMEDICAL INFORMATICS
ARIZONA STATE UNIVERSITY
SCOTTSDALE, AZ 85259

WANG, JUDY HUEI-YU, PHD *
ASSOCIATE PROFESSOR
DEPARTMENT OF ONCOLOGY
CANCER PREVENTION AND CONTROL PROGRAM
LOMBARDI COMPREHENSIVE CANCER CENTER
GEORGETOWN UNIVERSITY
WASHINGTON, DC 20007

WELLS, KRISTEN JENNIFER, MPH, PHD *
ASSOCIATE PROFESSOR
DEPARTMENT OF PSYCHOLOGY
SAN DIEGO STATE UNIVERSITY
SAN DIEGO, CA 92120

WILLIAMS, KRISTINE N, BSN, MS, PHD *
E JEAN HILL PROFESSOR
SCHOOL OF NURSING
UNIVERSITY OF KANSAS MEDICAL CENTER
KANSAS CITY, KS 66160

YEO, SEONAE, PHD *
PROFESSOR
SCHOOL OF NURSING
UNIVERSITY OF NORTH CAROLINA AT CHAPEL HILL
CHAPEL HILL, NC 27599

ZUNIGA, JULIE ANN, MSN, PHD *
ASSISTANT PROFESSOR
SCHOOL OF NURSING
UNIVERSITY OF TEXAS AT AUSTIN
AUSTIN, TX 78712

MAIL REVIEWER(S)

KOPYCKA-KEDZIERAWSKI, DOROTA TERESA, DDS, MPH
ASSOCIATE PROFESSOR
EASTMAN INSTITUTE FOR ORAL HEALTH
DIVISION OF COMMUNITY DENTISTRY
AND ORAL DISEASE PREVENTION
UNIVERISTY OF ROCHESTER
ROCHESTER, NY 14620

TANTISIRA, KELAN G, MD, MPH
ASSOCIATE PROFESSOR
DEPARTMENT OF MEDICINE
BRIGHAM AND WOMEN'S HOSPITAL
BOSTON, MA 02115

SCIENTIFIC REVIEW OFFICER

FORDYCE, LAUREN, PHD
SCIENTIFIC REVIEW OFFICER
CENTER FOR SCIENTIFIC REVIEW
NATIONAL INSTITUTES OF HEALTH
BETHESDA, MD 20892

EXTRAMURAL SUPPORT ASSISTANT

BUTLER, SEAN
LEAD - EXTRAMURAL SUPPORT ASSISTANT
CENTER FOR SCIENTIFIC REVIEW
NATIONAL INSTITUTE OF HEALTH
BETHESDA, MD 20892

* Temporary Member. For grant applications, temporary members may participate in the entire meeting or may review only selected applications as needed.

Consultants are required to absent themselves from the room during the review of any application if their presence would constitute or appear to constitute a conflict of interest.
